# Resting-state functional brain connectivity in a predominantly African-American sample of older adults: exploring links among personality traits, cognitive performance, and the default mode network

**DOI:** 10.1017/pen.2020.4

**Published:** 2020-03-27

**Authors:** Nicole T. Crane, Jessica M. Hayes, Raymond P. Viviano, Tim Bogg, Jessica S. Damoiseaux

**Affiliations:** 1 Institute of Gerontology, Wayne State University, Detroit, MI, USA; 2 Department of Psychology, Drexel University, Philadelphia, PA, USA; 3 Department of Psychology, Wayne State University, Detroit, MI, USA

**Keywords:** Openness, Big Five traits, Cognitive aging, Verbal fluency, Neuroimaging

## Abstract

The personality traits of neuroticism, openness, and conscientiousness are relevant factors for cognitive aging outcomes. The present study examined how these traits were associated with cognitive abilities and corresponding resting-state functional connectivity (RSFC) of the default mode network (DMN) in an older and predominantly minority sample. A sample of 58 cognitively unimpaired, largely African-American, older adults (*M* age = 68.28 ± 8.33) completed a standard RSFC magnetic resonance imaging sequence, a Big Five measure of personality, and delayed memory, Stroop, and verbal fluency tasks. Personality trait associations of within-network connectivity of the posterior cingulate cortex (PCC), a hub of the DMN, were examined using a seed-based approach. Trait scores were regressed on cognitive performance (delayed memory for neuroticism, Stroop for conscientiousness, and verbal fluency for openness). Greater openness predicted greater verbal fluency and greater RSFC between the PCC and eight clusters, including the medial prefrontal cortex, left middle frontal gyrus, and precuneus. Greater PCC–precuneus connectivity predicted greater verbal fluency. Neuroticism and conscientiousness did not significantly predict either cognitive performance or RSFC. Although requiring replication and elaboration, the results implicate openness as a contributing factor to cognitive aging via concomitant cognitive performance and connectivity within cortical hubs of the DMN and add to the sparse literature on these variables in a diverse group of older adults.

Meta-analytic and prospective research findings have shown the personality traits of neuroticism (anxious versus calm), conscientiousness (responsible versus careless), and openness (exploratory versus incurious) are consistent predictors of cognitive aging outcomes. Greater neuroticism has been associated with increased risk for Alzheimer’s disease (AD) (Terracciano et al., 2014), as well as poorer cognitive function, a steeper rate of cognitive decline in older adults, and an increased risk of mild cognitive impairment, a potential precursor to AD (Chapman et al., 2012; Kuzma, Sattler, Toro, Schönknecht, & Schröder, 2011; Luchetti, Terrcciano, Stephan, & Sutin, 2015). Lower levels of conscientiousness and openness have been associated with lower cognitive performance in older age, a faster rate of age-related cognitive decline, and increased risk of mild cognitive impairment, as well as AD (Chapman et al., 2012; Luchetti et al., 2015; Terracciano et al., 2014; Wilson, Schneider, Arnold, Bienias, & Bennett, 2007). Despite the associations with patterns of cognitive aging and related morbidity, there is a comparative paucity of evidence demonstrating how linkages between personality traits and markers of aging-related cognitive performance might be informed, in part, by shared neural systems.

Prior work exploring neural markers of personality traits has focused on structural or functional correlates of personality in general adult samples. The extant literature shows mixed results and personality traits have been linked to differences in structure and function of a variety of non-converging brain regions. Furthermore, these studies are primarily comprised of convenient, nonrepresentative samples that generally consist of younger or middle-aged adults of European-American descent with a high socioeconomic status (Falk et al., 2013). Therefore, there is a push for the field of neuroscience to use more diverse samples to enhance understanding of the brain mechanisms at play in a broader population. Currently, there are few investigations into how personality relates to neural systems relevant to aging within a minority population. With an urban sample consisting of 79% African-American participants, the present study afforded the opportunity to investigate interrelations among personality traits, cognitive abilities, and neural function within a largely minority group.

Previous work focusing on the relationships between personality and structural brain changes (e.g., volume loss) in older adult samples has assessed the extent to which structural brain atrophy that occurs with age was associated with individual differences in personality traits. Jackson, Balota and Head (2011) found that greater neuroticism was related to smaller volumes in cerebral gray matter and in the ventrolateral prefrontal, dorsolateral prefrontal (dlPFC), and orbitofrontal (OFC) cortices. Neuroticism also showed an interaction with age, whereby those greater in neuroticism showed a larger volumetric decrease in cerebral white matter with age. Conscientiousness was positively correlated with OFC volume in older adults; and individuals low in conscientiousness showed greater decrease in white matter, amygdala, and parahippocampal volumes with age. Wright, Feczko, Dickerson and Williams (2007) showed greater neuroticism was related to decreased thickness in the lateral superior and inferior frontal cortex among older adults. In another prospective study, greater neuroticism was associated with smaller OFC, dlPFC, and rolandic operculum regions; greater openness was related to larger frontopolar and smaller OFC and insular regions; and greater conscientiousness was associated with larger dlPFC and smaller frontopolar cortices (Kapogiannis, Sutin, Davatzikos, Costa, & Resnick, 2013).

While associations between personality traits and structural (i.e., volumetric) variation among older adults are informative, prior research has found functional connectivity as a premorbid biomarker of cognitive decline that occurs prior to or in the absence of structural changes (Hedden et al., 2009; Wierenga & Bondi, 2007). Indeed, disruptions in functional connectivity have been shown to precede structural changes and atrophy in the AD continuum (Gili et al., 2011), implicating functional measures as particularly informative when investigating brain changes in older age.

Research has investigated functional neural correlates of personality, and some has attempted to examine variations in brain networks that are not tied to a specific operationalization of a given task paradigm. For example, Liu et al. explored personality and resting-state functional connectivity (RSFC), which assesses regional brain interactions when participants are at wakeful rest (Liu, Kohn, & Fernández, 2019). Liu found unique patterns of RSFC specifically associated with each of the five-factor personality traits and utilized RSFC to successfully predict individual-level responses to a personality inventory. Other recent research investigated whether RSFC can reliably predict individual personality scores (Dubois, Galdi, Han, Paul, & Adolphs, 2018). This work showed openness was the only trait that could be reliably predicted based on RSFC patterns.

In their work, Sampaio et al. focused on personality and a specific RSFC network called the default mode network (DMN) (Sampaio, Soares, Coutinho, Sousa, & Gonçalves, 2014). Across individuals, the DMN is made up of functionally connected regions that show greater activation during rest than during cognitive tasks (Greicius, Krasnow, Reiss, & Menon, 2003). This network activates while the mind wanders, shows strong consistency across subjects (Damoiseaux et al., 2006), and includes the posterior cingulate cortex (PCC), medial prefrontal cortex (mPFC) (anterior cingulate cortex), precuneus, inferior parietal cortex, and parahippocampal gyrus (Greicius et al., 2003) among its major nodes. Sampaio et al. proposed that DMN connectivity is particularly relevant to personality because such activity is a trans-situational, trait-like mode of brain functioning, and could therefore inform, in part, the neural nature of individual differences in personality expression. In an adult sample, Sampaio et al. (2014) showed greater extraversion and agreeableness were associated with greater activity in the midline DMN regions, such as the precuneus and mPFC, while neuroticism, openness, and conscientiousness were correlated with parietal cortices of the DMN. These results implicate differential levels of DMN activity as functional correlates of trait expression. Beaty et al. (2016) also explored DMN connectivity and personality. Openness emerged as the most relevant trait for DMN function, accounting for 18% of the processing efficiency of the DMN. Combined, these results associating RSFC and personality metrics support the notion that functional connectivity is an important and relevant brain marker to consider when exploring a potential brain–personality correlate.

Although this initial evidence is notable, associations between personality traits and resting connectivity among older adults remain unclear. This is a particularly relevant omission for the study of cognitive aging and decline because decreased connectivity within the DMN has been observed in both typical aging (Andrews-Hanna et al., 2007; Damoiseaux et al., 2007; Sambataro et al., 2010) and pathological aging (Binnewijzend et al., 2012; Damoiseaux, Prater, Miller, & Greicius, 2012; Rombouts, Barkhof, Goekoop, Stam, & Scheltens, 2005). In this way, DMN connectivity is a candidate neural marker for age-related decline. Moreover, because personality traits – especially neuroticism, conscientiousness, and openness – are associated with age-related cognitive performance, DMN RSFC is the focus of our study and may represent a set of common neural factors linking the related expression of personality traits and markers of cognitive aging. The PCC, a major hub of the DMN (Greicius et al., 2003), was the *a prioi* region of interest (ROI) for our seed-based correlational analysis. The PCC has been isolated as a key component of a posterior DMN subsystem that shows network disruption prior to any structural changes associated with pathological aging (i.e., amyloid burden). This has led to a theory known as the cascade network failure hypothesis that stipulates alterations in this posterior DMN system, including the PCC, are the beginning of a cascade of neurodegeneration (Jones et al., 2015). Therefore, the PCC is of particular relevance when investigating DMN disruptions in older adults.

The aim of the present study was to evaluate the relationships between personality traits, cognitive performance, and functional connectivity in an older, largely African-American, sample. Because neuroticism, conscientiousness, and openness have emerged as important traits to consider when evaluating cognitive decline and brain measures in older age, as outlined above, they represented the focus of the present study. Extraversion and agreeableness show mostly non-convergent and null results in regard to cognitive decline and the aging brain (see Chapman et al., 2012; Jackson et al., 2011; Kapogiannis et al., 2013; Kuzma et al., 2011; Liu et al., 2019; Luchetti et al., 2015; Terracciano et al., 2014; Wright et al., 2007). Because of the limited evidence to inform hypotheses surrounding extraversion and agreeableness, they were not included in this investigation.

The first goal of the present work was to examine associations between neuroticism, conscientiousness, and openness and cognitive abilities in older adults. To do so, we identified cognitive outcomes of interest for each personality trait based on prior associations reported in the literature. Previous work has shown negative associations between delayed memory ability and neuroticism (Luchetti et al., 2015), where those greater in neuroticism recalled fewer words after a 5-min delay. For conscientiousness, the extant literature shows an association between facets of conscientiousness and impaired executive function abilities (including response inhibition) (Kumar, Yadava, & Sharma, 2016). Lastly, research shows that greater openness is predictive of greater verbal fluency ability (Graham & Lachman, 2014; Sutin et al., 2011). Based on these previously observed associations and to minimize the number of variables included in our analyses, we selected delayed memory as the relevant cognitive marker for neuroticism, response inhibition for conscientiousness, and verbal fluency for openness. We hypothesized that lower neuroticism, greater openness, and greater conscientiousness would predict greater cognitive performance in delayed memory, verbal fluency, and response inhibition tasks, respectively.

The second goal was to investigate the RSFC of a cortical hub of the DMN network to examine whether DMN RSFC represented a neural correlate of trait associations. We expected that neuroticism would negatively correlate with DMN connectivity, as greater neuroticism is a risk factor for aging effects. Furthermore, we expected that conscientiousness and openness would positively correlate with DMN connectivity, acting as potential protective factors against age-related network disruption.

## 1. Methods

### 1.1 Participants

Participants were healthy older adults between 50 and 85 years of age recruited from the metropolitan Detroit area. Exclusion criteria included left-handedness, neurological or psychological disorders, head or brain injury, cardiovascular disease, radiation or chemotherapy for cancer treatment, dementia, magnetic resonance imaging (MRI) contraindications, and regular or prolonged psychoactive medication use. Sample size was bound by recruitment feasibility from a largely minority population. Data were available for 66 potential participants. Eight were excluded due to incomplete or high motion MRI data, scores below 25 on the Mini-Mental Status Examination (MMSE) (Folstein, Folstein, & Mchugh, 1975), or for being below the age of 50 years. The remaining sample consisted of 58 participants for the trait and cognitive ability analyses (*M* age = 68.28 years, *SD* = 8.33) and 50 for the imaging analyses (*M* age = 67.78 years, *SD* = 8.47). In order to maximize data retention, listwise deletion for missing data was performed for each analysis individually. Participants were predominantly female (77.59%) and African-American (79.31%). Adding to the novelty of this sample, about 60% of our participants came from significantly distressed communities, scoring greater than 80 out of 100 (most distressed) on the Distressed Communities Index (DCI) score, which is a composite score summarizing the vitality of communities (available at eig.org/dci). The study sample represents a majority minority and high economic stress group that often goes understudied in neuroscience.

All participants scored 25 or greater on the MMSE (*M* = 29, *SD* = 1.67), scored no less than 1.5 *SD* below the normative mean on Wechsler Memory Scale – IV (WMS-IV) indices (Wechsler, 2009), and/or were cleared by clinical assessment as cognitively unimpaired. Average participant IQ, as measured by the Wechsler Abbreviated Scale of Intelligence-II, was 97.76 (*SD* = 11.22), which falls in the “low average” range based on scoring protocol (Wechsler, 2011). Participants completed a variety of questionnaires, including assessments of personality and a neuropsychological test battery. Participants completed the MRI protocol at the Wayne State University Magnetic Resonance Research Facility. The research protocol and materials were approved by the Wayne State University Institutional Review Board and all participants provided informed consent prior to participation.

### 1.2 Personality trait and cognitive measures

Personality traits were assessed with the 44-item Big Five Inventory (John, Naumann, & Soto, 2008; John & Srivastava, 1999). Participants indicated the extent to which a list of statements applied to them on a scale from 1 (Disagree Strongly) to 5 (Agree Strongly). Neuroticism was assessed based on eight items (i.e., “Is depressed, blue”). Conscientiousness was assessed on nine items (i.e., “Does a thorough job”). Openness was assessed on 10 items (i.e., “Is original, comes up with new ideas”). Scaled scores were calculated for each trait by calculating the mean of its relevant items. Alpha reliabilities were acceptable at .795 for neuroticism, .781 for conscientiousness, and .745 for openness.

Participants also completed the adult battery of the WMS-IV. As noted above, the WMS-IV delayed memory index, an assessment of delayed memory functioning for words and pictures after a 20–30-min delay, was selected based on prior reported relations between delayed memory ability and neuroticism. The delayed memory index is the average of an individual’s proportional scores (raw score divided by possible score) on the relevant delayed memory subtasks. Participants also completed a categorical verbal fluency task where they named all the animals they could think of in 1 min and then all the occupations they could think of in 1 min. Verbal fluency scores were the sum of correct item instances across both prompts. Participants also completed the Stroop task (Stroop, 1935) to evaluate response inhibition. Stroop ratio scores were the time spent on the Stroop trial divided by the time spent on the color-only trial. Linear regression assessed the relationship between personality dimensions and cognitive performance after controlling for age and sex as covariates. Shapiro–Wilk tests of normality were used to assess the distribution of all relevant variables. If variables were found to be non-normally distributed, regression analyses were run with both transformed variables (that address normality issues) as well as original, raw data to compare results.

### 1.3 MRI data acquisition and processing

Images were acquired on a 3-Tesla Siemens Magnetom Verio full-body scanner (Siemens Medical AG, Erlangen, Germany) with a 32-channel head coil. Anatomical T1-weighted sequence: magnetization-prepared rapid gradient-echo sequence with 176 slices collected parallel to the bicommissural line, repetition time (TR) = 1680 ms, echo time (TE) = 3.51 ms, inversion time = 900 ms, flip angle = 9.0°, pixel bandwidth = 180 Hz/pixel, GeneRalized Autocalibrating Partially Parallel Acquisitions (GRAPPA) acceleration factor phase encoding (PE) = 2, field of view (FOV) = 256 mm, matrix size = 384 × 384, and voxel size = 0.7 × 0.7 × 1.3 mm. Structural scan duration was 6 min. Resting-state functional sequence: T2*-weighted echo-planar imaging sequence with 37 slices parallel to bicommissural line, 200 image volumes, TR = 2200 ms, TE = 30 ms, flip angle = 80°, pixel bandwidth = 2232 Hz/pixel, GRAPPA acceleration factor PE = 2, FOV = 220 mm, matrix size = 80 × 80, voxel size = 2.8 mm isotropic. Resting-state scan duration was 8 min.

We completed image processing and analysis with FMRIB Software Library tools (FSL 5.0.8; https://fsl.fmrib.ox.ac.uk; Jenkinson, Beckmann, Behrens, Woolrich, & Smith, 2012). Both structural and functional image preprocessing included removal of nonbrain voxels (Smith, 2002). Further resting-state image preprocessing included removal of the first five image volumes to account for early field inhomogeneities, motion correction (Jenkinson, Bannister, Brady, & Smith, 2002), spatial smoothing (6 mm full width at half maximum), 4D-grand mean scaling, independent component analysis-based Automatic Removal of Motion Artifacts (ICA-AROMA) (Pruim et al., 2015), and global signal regression. We applied spatial smoothing and global signal regression to further increase the signal-to-noise ratio in our data as we still observed some global noise after the other preprocessing steps. Images remained in native space.

We conducted a seed-based correlation analysis with the PCC as our *a priori* ROI based on its relevancy in DMN disruption and the cascade network hypothesis (Jones et al., 2015). We used the PCC coordinates from Fox et al. (2005), which showed a strong correlation with relevant task-negative regions (i.e., DMN). We generated participant-specific ROIs in native space based on the semi-automated method outlined in Viviano et al. (2019). The method corrects for individual variability in brain region size and location, ensuring correct placement of the ROI in native space. In short, for each participant, we transformed the PCC coordinate from standard space to individual T1-structural space and visually inspected the transformation for accurate placement. We then generated a spherical ROI with 6-mm diameters and masked it with binarized gray-matter segmentation images. Next, we transformed the PCC ROI to the functional image space mapped to the T1-weighted image and extracted the average resting-state time-course.

First-level FMRI Expert Analysis Tool (FEAT) v6.00 analysis (Woolrich, Ripley, Brady, & Smith, 2001) assessed PCC time series correlation across the whole brain, producing a contrast image showing voxels whose time series significantly correlated with the PCC. We transformed individual contrast images to Montreal Neurologic Institute standard space with FMRIB’s Linear Image Registration Tool (Jenkinson et al., 2002; Jenkinson & Smith, 2001) and then merged subject-level images into a single 4D group-level image for nonparametric higher-level analyses with FSL Randomise (Winkler, Ridgway, Webster, Smith, & Nichols, 2014). Prior to running Randomise, a one-sample t-test generated a group-level PCC connectivity map. After multiplication with a gray matter mask, this network map allowed us to focus on regions with strong connectivity to the PCC. Next, we ran Randomise with 5000 permutations to assess the relationship between PCC RSFC and personality while controlling for age and sex. Separate models evaluated neuroticism, conscientiousness, and openness. We first determined significance based on a stringent *p* < .05 family-wise error-corrected threshold. If that was not met, due to the exploratory nature of this analyses (i.e., in a minority sample) and a relatively small sample, we also considered a more lenient threshold of *p* < .01 uncorrected.

## 2. Results

### 2.1 Associations among age, sex, racial/ethnic background, and cognitive performance and personality traits

A correlation matrix for all study variables is shown in Table 1. Older age was associated with lower verbal fluency performance (*r* = −.394, *p* = .002) and poorer performance on the WMS-IV delayed memory index (*r* = −.527, *p* < .001), but was not associated with Stroop performance (*r* = .130, *p* = .341). Age was negatively correlated with openness (*r* = −.291, *p* = .026), but uncorrelated with neuroticism (*r* = −.030, *p* = .821) and conscientiousness (*r* = −.058, *p* = .668). An independent samples t-test, equal variance not assumed, showed that males (*M* = 2.13, *SD* = 0.66) outperformed females (*M* = 2.85, *SD* = 1.39) on the Stroop task, *t*(43.26) = 2.56, *p* = .014. In addition, females (*M* = 0.48, *SD* = 0.11) outperformed males (*M* = 0.41, *SD* = 0.11) on the WMS-IV delayed memory index, *t*(54) = 2.04, *p* = .046. There were no sex differences in verbal fluency performance, *t*(55) = −0.91, *p* = .37. There was no significant difference between African-American participants and European-American participants on any of the measures. Shapiro–Wilk tests revealed that both Stroop ratio scores, W(54) = 0.86, *p* < .01, and conscientiousness scores, W(54) = 0.93, *p* < .01, significantly diverged from normality. Therefore, analyses that included these variables used both raw and transformed variables (log-transformed for Stroop and reflection-and-square-root-transformed for conscientiousness). Because the results did not differ between these approaches, raw data results are reported for ease of interpretation.

**Table 1.** Pearson correlations among demographic, personality, and cognitive performance variables for all participants

Variables	1	2	3	4	5	6	7	8
1. Age	–							
2. Sex	−.093	–						
3. Race	.254	−.134	–					
4. Openness	−.291*	.092	.037	–				
5. Conscientiousness	−.058	−.044	−.069	.237	–			
6. Neuroticism	−.030	.085	−.168	−.052	−.577**	–		
7. Verbal fluency	−.394**	.121	.002	.361**	.217	.022	–	
8. Stroop ratio score	.130	−.237	.115	−.157	.202	−.146	−.130	–
9. Delayed memory index	−.527**	−.267*	−.176	.290*	.110	−.095	.505**	−.230

** Denotes significance at *p* < .01.

* Denotes significance at *p* < .05.

### 2.2 Associations between personality and cognitive measures

For openness, we found a significant relationship with verbal fluency performance. The model was significant and accounted for 18% of the variance (Adjusted *R* ^2^ = .181, *F*(3, 53) = 5.11, *p* < .01). Age and openness were significant predictors of fluency scores (age *β* = −0.31, *p* = .019; openness *β* = 0.26, *p* = .043). Sex was not a significant predictor. Neuroticism was not related to delayed memory performance. The overall model was significant and accounted for 35% of the variance (Adjusted *R* ^2^ = .350, *F*(3, 52) = 10.87, *p* < .001), but was driven by age and sex effects (age *β* = −0.56, *p* < .001; sex *β* = −0.31, *p* < .01). Neuroticism was not a significant predictor for delayed memory function, *β* = −0.08*, p* = .452. When assessing the association between conscientiousness and performance on the Stroop task, the model of age, sex, and conscientiousness was not significant (Adjusted *R* ^2^ = .054, *F*(3, 52) = 2.05, *p* = .118).

### 2.3 Personality, cognitive performance, and functional connectivity

**Figure 1.**
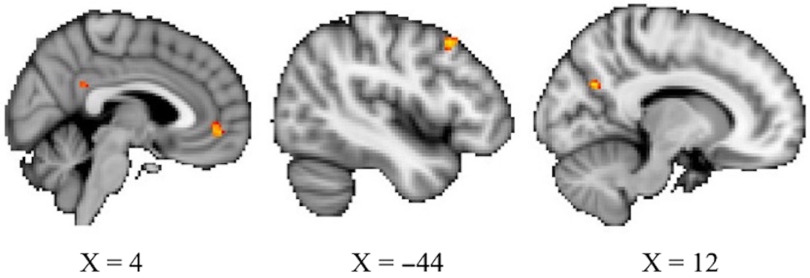
Three largest clusters whose RSFC with the PCC show a positive relationship with openness at *p* < .01, uncorrected. From left, the mPFC (41 voxels), left middle frontal gyrus (27 voxels), and precuneus (27 voxels).

Nonparametric permutation testing of brain imaging data revealed eight clusters whose functional connectivity with the PCC showed a significant positive relationship with openness while controlling for age and sex, at the more lenient threshold of *p* < .01 uncorrected. Those greater in openness had stronger connectivity between these regions and the PCC. The largest of these clusters were in the mPFC at 41 voxels, the left middle frontal gyrus at 27 voxels, and the precuneus at 27 voxels. Cluster locations and details are provided in Figure 1 and Table 2. Analyses did not show any significant associations between neuroticism or conscientiousness and PCC RSFC.

**Table 2.** Regions where RSFC with the PCC was associated with openness

Brain regions	Voxels	*p*-Max	*z*-stat	*X*	*Y*	*Z*
Paracingulate/medial prefrontal cortex	41	.002	2.91	4	50	−6
Left middle frontal gyrus	27	.001	3.01	−44	18	48
Precuneus	27	.001	3.00	12	−66	28
PCC	19	.004	2.70	8	−44	24
Right occipital	11	.006	2.54	44	−62	42
Left paracingulate/medial prefrontal cortex	10	.004	2.63	−10	42	−8
Left middle frontal gyrus	7	.004	2.60	−34	10	36
Frontal pole	4	.002	2.74	−14	66	−8

Coordinates are in MNI space.

**Figure 2.**
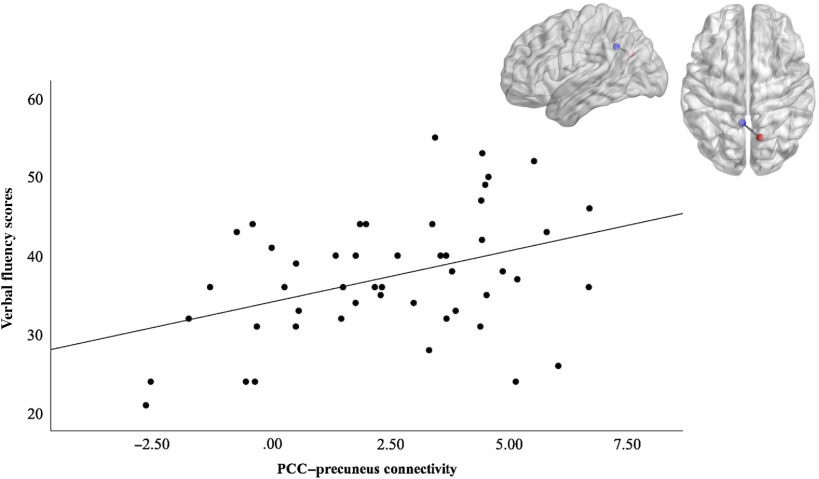
Illustration of the PCC ROI (blue) – precuneus (red) functional connectivity and its positive correlation with verbal fluency scores. The graph depicts the positive correlation between PCC-precuneus connectivity and verbal fluency performance to help illustrate the significant predictive effects of functional connectivity on fluency outcomes found in regression analyses (Adjusted *R^2^* = .171, *F*(2, 46) 5.96, *p* < .01).

Post hoc analysis on this finding evaluated clusters larger than 10 voxels to assess whether strength of the connectivity between these regions and the PCC, already shown to be associated with openness, also predicted cognitive performance. The clusters in the mPFC, left middle frontal gyrus, precuneus, PCC, and right occipital met the size threshold. We generated cluster masks and applied them to subject-level *z*-stat images to extract mean parameter estimates for nonzero voxels in the clusters. Linear regression analyses were conducted to assess the relationship between functional connectivity measures and verbal fluency performance. We included age as a covariate because older age was associated with lower connectivity between the PCC and the precuneus (*r* = −.433, *p* = .002). We did not include sex as a covariate in this analysis because it did not significantly relate to verbal fluency performance. Regression results showed a significant relationship between PCC – precuneus connectivity and verbal fluency scores, as seen in Figure 2. The overall model was significant and accounted for 17% of the variance (Adjusted *R* ^*2*^ = .171, *F*(2, 46) = 5.96, *p* < .01). Only PCC–precuneus connectivity was a significant predictor, *β* = 0.30, *p* = .044. Age was not a significant predictor. Regression results for an association between the other connections (between the PCC and mPFC, left middle frontal gyrus, PCC and right occipital) and verbal fluency were not significant.

## 3. Discussion

The primary goal of the present work was to investigate RSFC in the DMN as a concomitant neural correlate of associations between personality traits and cognitive performance in a sample of older, predominantly African-American adults. The results showed greater openness was associated with greater verbal fluency, as well as greater PCC RSFC within the DMN. Prior research implicated openness as a relevant trait in predicting cognitive aging trajectories. In the present work, those findings were replicated, in part, by showing a relationship between openness and cognitive function in older age. Verbal fluency indicates cognitive flexibility, and openness has been linked to enhanced cognitive flexibility (Ayotte, Potter, Williams, Steffens, & Bosworth, 2009), possibly due to increased creativity and verbal ability being key parts of openness (Sneed, McCrae, & Funder, 1998). Based on these characteristics, it follows that those high in openness could have increased ability to generate novel words and ideas in a verbal fluency task. Our findings replicate prior work in this area (Sutin et al., 2011) and imply that the association between openness and cognitive function also applies to a minority sample.

In addition to predicting cognitive performance, greater openness was also associated with increased RSFC of the PCC to eight other clusters in the brain. The three largest were in the mPFC, the left middle frontal gyrus, and the precuneus. The mPFC and precuneus are functionally connected to the PCC and are considered primary components of the DMN (Greicius et al., 2003). In the present study, greater openness was related to stronger connectivity among these DMN regions in older age, suggesting characteristics related to openness, that is curiosity, creativity, or imagination, could act as protective factors against the deleterious effects of aging via comparatively increased DMN connectivity. Individuals reporting greater openness exhibited stronger DMN connections, independent of age. This corroborates prior findings which also found openness, of all the traits in the five-factor model of personality, to be the trait most robustly associated with RSFC (Dubois et al., 2018) and DMN connectivity specifically (Beaty et al., 2016). As noted above, the majority of neuroimaging work focuses on convenience samples, so it is meaningful to extend these relationships between openness and RSFC to a more diverse group of older adults.

Post hoc analyses related the strength of these DMN connections associated with openness back to predicting behavior, as the extent of PCC–precuneus connectivity during rest predicted verbal fluency performance. Individuals with weaker PCC–precuneus connections tended to perform worse on verbal fluency tasks. Taken together, the findings suggest that DMN RSFC could be a potential neural correlate of the relationship between personality and cognitive performance, where low levels of openness predict decreased communication between DMN regions and decreased performance on task-relevant cognitive function. Note that during the post hoc analysis, we did explore adding openness as a predictor to the existing model of PCC–precuneus connectivity and age predicting verbal fluency. With openness in the model, none of the predictors were significant, even though the overall model was statistically significant. This is likely related to the shared variance of openness, PCC–precuneus connectivity, and age. The PCC–precuneus connectivity was specifically selected as a predictor in the model for verbal fluency due to its prior relation to openness in the initial analyses. Therefore, it follows that this functional connectivity and openness would have common variance in the prediction of verbal fluency performance and overlaps with our findings in the initial level of analyses.

Neuroticism and conscientiousness showed null results in any associations with cognitive ability (delayed memory or response inhibition, respectively) and PCC connectivity. This differs from some previous findings which show these traits relating to aging trajectories (Chapman et al., 2012; Kuzma et al., 2011; Luchetti et al., 2015; Terracciano et al., 2014; Wilson et al., 2007) and RSFC patterns (Adelstein et al., 2011; Aghajani et al., 2014; Hsu, Rosenberg, Scheinost, Constable, & Chun 2018; Kunisato et al., 2011). This could simply be due to lack of power in our study with a relatively small sample size. However, it could also be related to the uniqueness of our sample in that it was majority African-American which diverges from the groups studied in previous work. Therefore, it is possible that the relationships among these variables differ within a diverse sample of low-average IQ and from high-distressed communities, compared to what has been previously found in mostly highly educated samples of European ancestry. We did explore the effect of IQ and MMSE on these results by including these variables in the statistical models. We found that their inclusion did not meaningfully change the predictive value of the personality measures. Additional research is needed in this realm to address any potential population differences in the relationships between neuroticism/conscientiousness, DMN connectivity in age, and cognitive ability.

Further work could investigate potential mediators of the openness–PCC RSFC and openness–cognitive function relationships. Openness has been shown to be associated with general (e.g., need for cognition, absorption) and specific sensory and information-seeking (e.g., leisure time physical activity, health-related Internet search frequency) tendencies and behaviors (Bogg & Vo, 2014; DeYoung, Grazioplene, & Peterson, 2012; Wilson & Dishman, 2015). Presently, it is unclear whether the relationship between openness and aging outcomes (RSFC or cognitive performance) is a direct link or whether it is better explained through engagement in sensory, investigative, or exploratory activities, including high intellectual engagement or increased exercise, for example, both of which have been associated with greater levels of openness (McCrae & Costa, 1997; Rhodes, Courneya, & Jones, 2003). Next steps for this line of research also could include an examination of whether specific facets of openness (Soto & John, 2009) are more or less predictive of cognitive performance and PCC RSFC in order to further refine an understanding of the aspects of openness that are most informative of aging outcomes.

As noted, the diversity of our sample, which consisted of nearly 80% African-American participants, is a significant strength of the present study, given that most neuroimaging samples are primarily comprised of European-American participants. This work contributes to the much-needed body of literature on brain metrics in minority samples, in part, by showing null effects for differences in the study variables based on racial/ethnic background. However, it is important to interpret these and the other results in the context of the study limitations. The RSFC results presented did not withstand correction for multiple comparisons. Therefore, this work should be interpreted as preliminary findings in this unique, minority sample. Generally, additional work is needed to replicate these findings before drawing further conclusions regarding the robustness of the role of openness as it relates to PCC connectivity. Moreover, although adequately powered for moderate-to-large-sized effects, the sample size precluded reliable detection of small effects.

Despite the limitations, the present work indicated that greater levels of openness were associated with greater performance on a verbal fluency task, as well as increased connectivity of a primary cortical hub of the DMN. The results extend the findings of prior research by providing preliminary evidence for the role of openness in concomitant patterns of aging-related neural and cognitive expression among older adults and exploring how these relationships extend to an aging, largely African-American population.
